# Examination of the Pediatric Cervical Spine Under Anesthesia

**DOI:** 10.7759/cureus.64623

**Published:** 2024-07-15

**Authors:** Blake K Montgomery, Keith Orland, Troy A Wilson, William Chen, Otis C Shirley, Anand Segar, Antony J Field, Haemish A Crawford

**Affiliations:** 1 Orthopaedics, Starship Children’s Hospital, Auckland, NZL

**Keywords:** fluoroscopy, trauma, instability, cervical spine, pediatric

## Abstract

Cervical spine injuries in pediatric patients can have devastating consequences if not properly diagnosed. The standard workup for suspected cervical spine injuries includes cervical X-rays and a high-resolution CT. If suspicion still exists then a cervical MRI is obtained. When the cervical MRI shows ligamentous edema but is unable to determine the integrity of the ligaments then additional workup is needed. Often a flexion and extension lateral cervical X-ray can help determine ligament integrity in the non-sedated cooperative age-appropriate patient. For pediatric patients who are unable to perform the flexion and extension X-ray, we perform a dynamic fluoroscopic examination of the cervical spine under anesthesia. The patient is positioned in the supine position. The C-arm is positioned in the lateral position. The surgeon manually performs distraction, flexion, extension, and translation maneuvers while obtaining live fluoroscopy and assessing for signs of cervical instability. If cervical instability exists then the appropriate definitive treatment can be performed. If the cervical spine is stable then cervical immobilization can be discontinued.

## Introduction

The cervical spine is the most common location of spinal injury in pediatric patients and can result in devastating neurological injury if not properly identified and treated [1,2]. Additionally, pediatric patients have a higher chance of upper cervical injuries compared to adults which is attributed to their higher head-to-torso ratio, increased flexibility, and relatively weaker muscles. Making the diagnosis can be challenging in children due to the child’s age, level of cooperation, difficulty in visualizing non-ossified bone on imaging, accompanying injuries, and intubation status. When a cervical injury is suspected, cervical X-rays and a high-resolution CT should be obtained. If suspicion still exists, then a cervical MRI is obtained [3]. At our institution, patients who are not obtunded would get erect cervical imaging with flexion and extension views when possible. We do not perform examination under anesthesia (EUA) of the spine for patients who can be examined and can perform flexion and extension.

The cervical MRI is critical to evaluate the spinal cord, spinal canal, non-ossified cartilage structures, and the occipitocervical ligaments. While the cervical MRI is the best imaging technique to evaluate the cervical ligaments, it can fall short of determining the true integrity of the ligaments and ultimately the stability of the cervical spine. The integrity of the cervical ligaments may be particularly concerning in patients with significant head trauma and massive amounts of cervical edema.

Additional investigation is needed when the MRI is unable to determine if the ligamentous injury is severe enough to lead to cervical instability [4]. A flexion and extension lateral cervical X-ray can be obtained in the non-sedated cooperative age-appropriate patient [5]. Some pediatric patients, especially patients intubated and sedated for other injuries, are unable to comply with flexion and extension X-rays. A cervical orthosis can increase the risk of aspiration, increase intracranial pressure, and increase the chances of creating a pressure-related sore. For these reasons, every effort should be made to determine if the spine is stable in order to remove unnecessary cervical immobilization [6,7]. To determine cervical stability in patients who are unable to perform independent flexion and extension X-rays, we perform cervical EUA.

## Technical report

Case example

**Figure 1:**
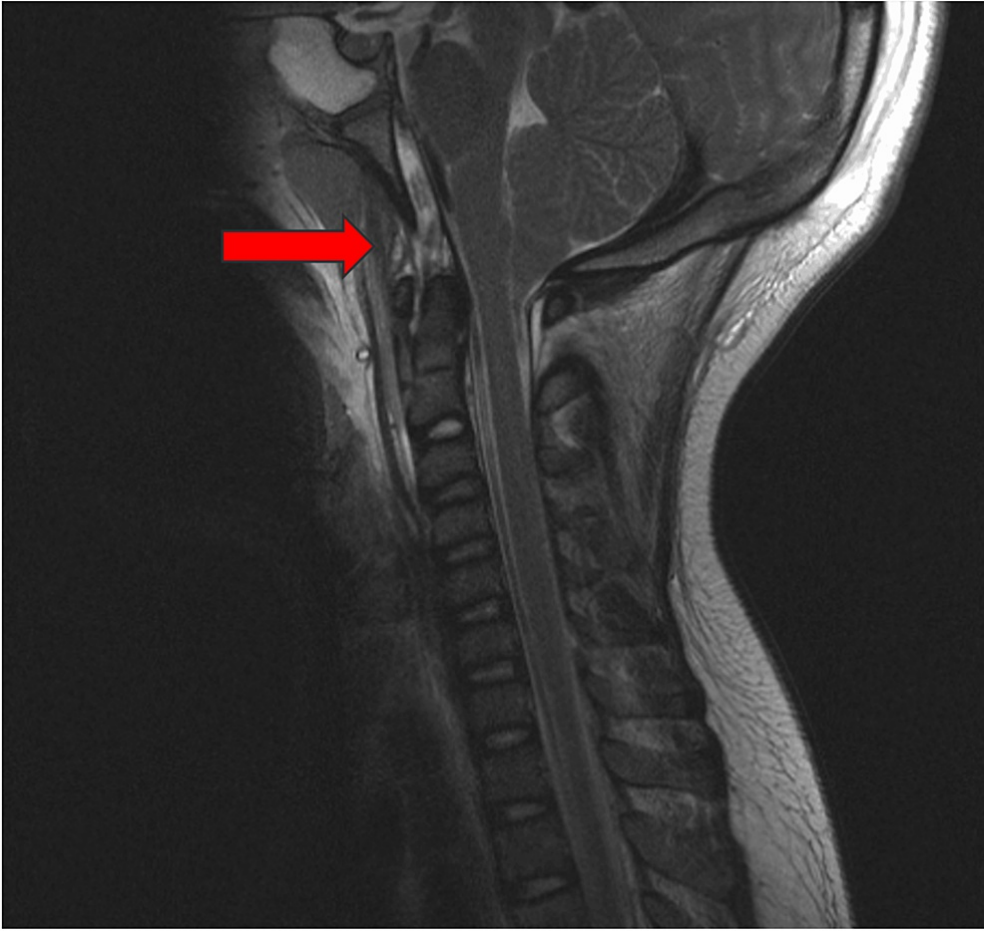
T2 MRI of a 9-year-old patient demonstrating questionable integrity of the ligamentous complex.

A 9-year-old patient who was struck by a car sustained multiple injuries requiring intubation. Cervical MRI demonstrated questionable integrity of the ligamentous complex (Figure 1). Cervical EUA demonstrated significant C0-C1 translation and C1-2 instability. The patient underwent occiput to C2 instrumented fusion.

Description of the method

**Figure 2:**
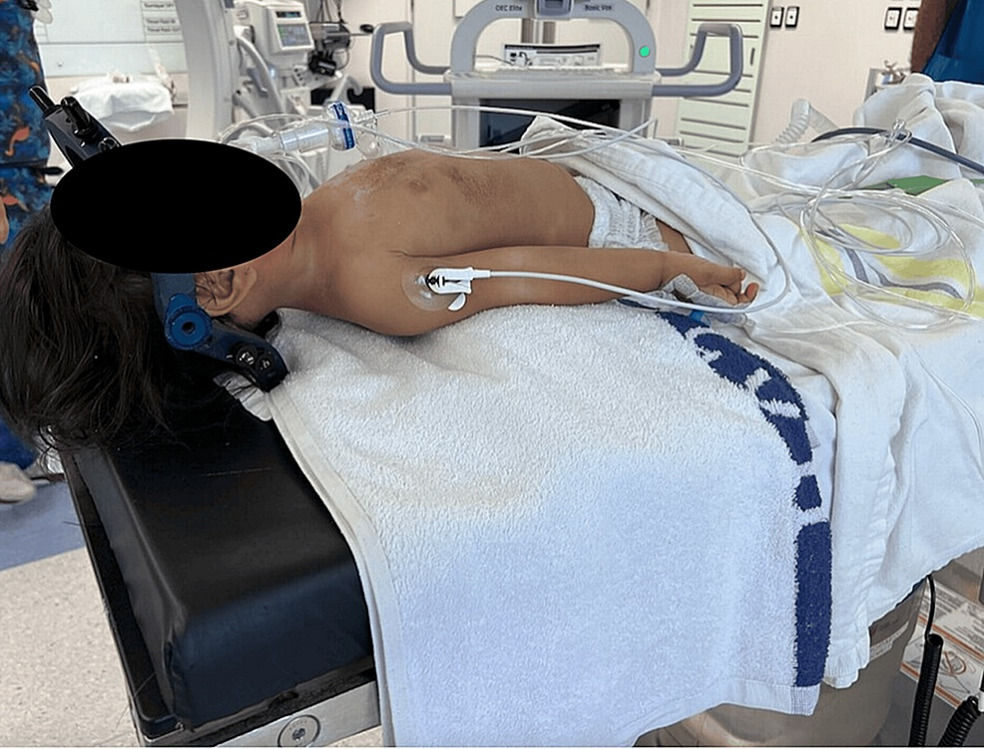
The patient is positioned supine with towels under the chest to allow for the neutral neck position (a patient from the case example).

Prior to the procedure, we have all the equipment prepared in order to address the potentially unstable cervical spine. This is usually a halo or instrumentation for fusion. We maintain cervical precautions and in-line traction while positioning the patient supine on a standard table. The size of the pediatric head forces the neck into flexion if the child is placed on a flat surface. Therefore, the chest is elevated on folded towels to allow the neck to rest in a neutral position (Figure 2).

**Figure 3:**
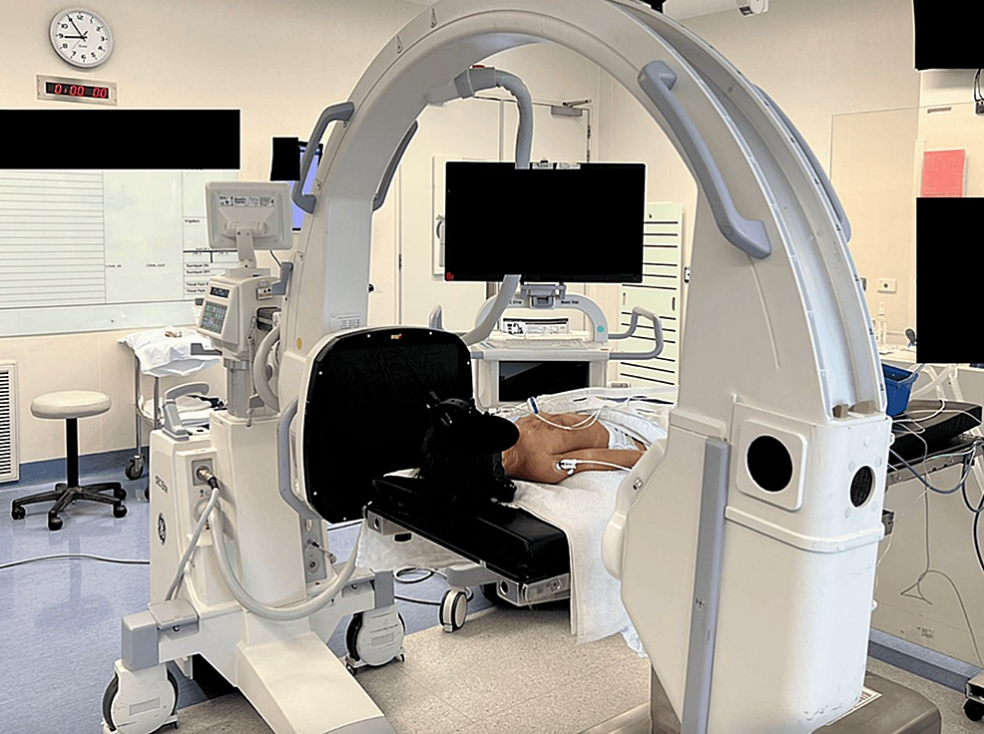
The C-arm is positioned for lateral X-rays. The monitor is positioned adjacent to the table so the surgeon has an unimpeded view of the screen during the examination (a patient from the case example).

**Figure 4:**
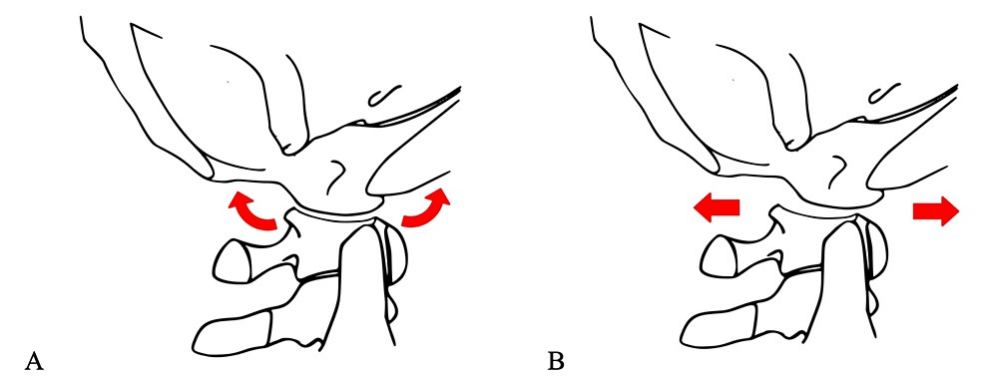
Diagram depicting normal rocking (A) and pathological translation (B) at the occipitocervical joint. Image Credit: Troy A. Wilson

The C-arm is positioned for lateral X-rays. The monitor is positioned next to the C-arm so that the surgeon can view the monitor while performing the exam (Figure 3). Lead personal protective equipment should be worn by the surgical team. This can include lead gloves worn by the surgeon performing the EUA. Next, dynamic distraction is performed by manually distracting the skull while obtaining live fluoroscopy. The examiner should pay close attention to the occiput in relation to C1 to ensure pathologic widening does not occur [8,9]. Additionally, tension should be appreciated in the entire cervical spine if the ligamentous complex is intact. Next, dynamic flexion and extension X-rays are obtained. Special attention should be paid to the C0-C1 joint, anterior Atlantodens interval, and the interspinous distance between the spinous processes. Lastly, anterior and posterior translation is performed. When the occiptocervical ligaments are intact the atlantoocciptal joint should rock and should not translate as the joint is elliptical in shape (Video 1, Figure 4). Occipitocervical translation occurs when capsular and ligamentous integrity is compromised. We do not use neuromonitoring during the EUA. While all of these dynamic assessments may be recorded by the radiographer, we typically record the live images with a mobile device for subsequent review.

**Video 1:** Cervical examination under anesthesia (EUA) Cervical EUA was performed under fluoroscopy with special attention paid to the CO/C1 joint showing normal distraction, normal flexion and extension, normal translation, and pathological anteroposterior (AP) translation.

## Discussion

Comparison to other methods

Our described method of EUA has many similarities to other methods previously described. For adult patients, dynamic fluoroscopy has been described to help determine cervical stability in comatose patients [10,11]. In adults, CT has obviated the need to perform dynamic fluoroscopy; however, pediatric injuries are very different than adult injuries and should not be lumped into the same category without proper investigation [12,13]. For pediatric patients, Anderson et al. depict dynamic cervical X-rays within their cervical spine clearance protocol [14]. While the authors mention dynamic flexion-extension cervical X-rays, they do not describe a detailed technique. To the best of our knowledge, this is the first described technique of dynamic fluoroscopy to assess cervical stability in pediatric patients. We have performed dynamic fluoroscopy on over 30 patients.

## Conclusions

In summary, we have described a technique to assess for upper cervical instability in pediatric patients using dynamic fluoroscopy. The primary advantage of this technique is determining cervical stability status when the MRI is inconclusive. Cervical immobilization increases the risks of adverse events and this technique may decrease the time of unnecessary cervical immobilization. The disadvantage of this technique is it requires a general anesthetic and exposes the patient and the surgeon to radiation.
